# Non-pulmonary arterial involvement in Behçet’s disease: a multicenter retrospective case series

**DOI:** 10.55730/1300-0144.6206

**Published:** 2026-02-16

**Authors:** Özge KARAKÖK, Duygu KERİM, Şerife ÇOŞKUN SAĞIRKAYA, Gamze AKKUZU, Burcu Ceren ULUDOĞAN, Gezmiş KİMYON, Hasan KOCAAYAN, Senar ŞAN, Özkan URAK, Gökçe KENAR, Ayşe CEFLE, Servet AKAR, Nazife Şule YAŞAR BİLGE, Cemal BES, Şükran ERTEN, Ahmet OMMA, Gökhan KESER, Kenan AKSU, Tulin ERGUN, Rafi Haner DİRESKENELİ, Fatma ALİBAZ ÖNER

**Affiliations:** 1 Division of Rheumatology, Department of Internal Medicine, Faculty of Medicine, Marmara University, İstanbul, Turkiye; 2 Division of Rheumatology, Department of Internal Medicine, Faculty of Medicine, Ege University, İzmir, Turkiye; 3 Division of Rheumatology, Department of Internal Medicine, Bilkent City Hospital, Ankara, Turkiye; 4 Division of Rheumatology, Department of Internal Medicine, Başaksehir Çam ve Sakura City Hospital, İstanbul, Turkiye; 5 Division of Rheumatology, Department of Internal Medicine, Faculty of Medicine, Eskisehir Osmangazi University, Eskişehir, Turkiye; 6 Division of Rheumatology, Department of Internal Medicine, Faculty of Medicine, Mustafa Kemal University, Hatay, Turkiye; 7 Division of Rheumatology, Department of Internal Medicine, Faculty of Medicine, Katip Celebi University, Izmir, Turkiye; 8 Division of Rheumatology, Department of Internal Medicine, Faculty of Medicine, Kocaeli University, Kocaeli, Turkiye; 9 Division of Rheumatology, Department of Internal Medicine, Faculty of Medicine, Dokuz Eylül University, İzmir, Turkiye; 10 Department of Dermatology, Faculty of Medicine, Marmara University, İstanbul, Turkiye

**Keywords:** Behçet’s disease, arterial involvement, arterial aneurysm, arterial thrombosis

## Abstract

**Background/aim:**

In Behçet’s disease (BD), arterial involvement represents a significant clinical challenge associated with serious complications. The present study evaluates the characteristic features, affected vessels, lesion types, treatments, and outcomes in BD patients with nonpulmonary arterial involvement.

**Materials and methods:**

Included in this retrospective study were 65 BD patients whose interventions and clinical courses were accessed from medical records. The treatments administered included immunosuppressive (IS) therapies and surgical/endovascular procedures. Fisher’s exact and t-test/Mann–Whitney U test were used for statistical analysis.

**Results:**

Fifty-nine of the patients (90.8%) were male. The median (Q1–Q3) follow-up was 4 (2–12.8) years. The most commonly affected vessels were the aorta (n = 21, 32.3%) and the femoral artery (n = 19, 29.2%). Aneurysm (n = 50, 63.2%) and thrombosis (n = 18, 22.7%) were the predominant lesions; 14 (21.5%) patients experienced arterial involvement while receiving IS therapy; and 11 (16.9%) had cardiac involvement. Cyclophosphamide and azathioprine were the most commonly used IS agents. Among the 39 (60%) patients who underwent surgical and/or interventional procedures, 32 (82%) had successful outcomes. A second arterial event was experienced by 15.3% of the sample during follow-up, and a third by 3%. Active disease was noted in nine (13.8%) patients at the time of their last visit, and of the six (9.2%) that died, most had both arterial and cardiac involvement.

**Conclusion:**

Arterial involvement in BD mostly presents with aneurysms, especially in men. Surgical interventions in addition to IS therapies are required in approximately 50% of cases. As can be understood from the 15.3% relapse rate in the present study, arterial events are not uncommon, underlining the need for long-term monitoring and sustained IS treatment. Of the 9.2% of cases who died, most had both arterial and cardiac involvement.

## 1. Introduction

Behçet’s disease (BD) is a complex multisystem disorder characterized by recurrent oral and genital ulcers, along with a range of other manifestations, including ocular, vascular, gastrointestinal, and neurological involvement. The clinical manifestations of BD are markedly heterogeneous and may vary significantly according to sex, ethnicity, age at onset, and geographic origin. The onset of BD usually occurs in the third decade of life and affects both sexes equally, but tends to follow a more severe course in men [1]. Diagnosis is primarily clinical due to the absence of specific laboratory tests, and the disease typically follows a relapsing–remitting course.

BD, classified as a variable-vessel vasculitis, is uniquely characterized by the involvement of both arteries and veins of all sizes, with a distinct tendency for aneurysm formation [2, 3]. Vascular involvement is seen in about one-third of cases, and is more common and more severe in younger male patients [4]. Deep vein thrombosis (DVT) is the most common form of vascular involvement in BD. While arterial complications are less frequent, occurring in approximately 3%–5% of patients, they remain a distinctive feature of BD, which is among the few chronic inflammatory disorders capable of inducing aneurysms in peripheral, visceral, and pulmonary arteries [5]. Despite its low incidence, arterial involvement in BD significantly contributes to morbidity and mortality, primarily through such severe complications as arterial occlusion and aneurysmal rupture. Aneurysm formation is more common than thrombosis, occurring most often in the abdominal aorta, and the pulmonary, femoral, popliteal, and carotid arteries [6]. Immunosuppressive (IS) therapy is sometimes used in combination with vascular surgery to control the disease. The surgical and medical management of BD differs from that of atherosclerotic aneurysms owing to the unique characteristics of the disease [7].

We conducted a retrospective review of 65 Turkish BD patients with nonpulmonary arterial involvement to fill the gaps in the literature related to the clinical features, course, and outcomes of the disease, clarifying the initial clinical symptoms, lesion locations, and treatment protocols.

## 2. Materials and methods

### 2.1. Study population and data collection

Included in this multicenter retrospective study were 65 patients who underwent treatment at nine rheumatology centers in Türkiye between January 1998 and December 2024. Patients with isolated pulmonary artery involvement were excluded. All 65 patients fulfilled the International Study Group (ISG) criteria or the International Criteria for Behçet’s Disease (ICBD)[8, 9].

Demographic data, the clinical characteristics of the first vascular event and subsequent relapses, treatment protocols, and details of any complications were acquired retrospectively from patient records. Arterial disease was diagnosed either by Doppler scan, echocardiography, computed tomography (CT) angiography, or magnetic resonance (MR) angiography.

Criteria for disease activity included: (1) the onset or worsening of systemic and/or vascular symptoms not attributable to another condition; (2) elevation of acute-phase reactants (erythrocyte sedimentation rate (ESR) and C-reactive protein (CRP)); and (3) the appearance of one or more vascular lesions in previously unaffected territories and/or progression of preexisting lesions. Remission was defined as the resolution of clinical signs, laboratory findings, and, imaging evidence (if available) of active disease, together with the absence of new vascular lesions and the lack of progression of existing vascular involvement.

Surgical/interventional success was assessed retrospectively, defined as symptomatic improvement documented in patient records, and supported by imaging findings such as graft/stent patency or stability of arterial lesions, when available. Due to the retrospective study design, no standardized radiological or objective success criteria could be applied.

The study protocol was approved by the Institutional Review Board of Marmara University Faculty of Medicine (Protocol no: 09.2023.1308), and the study was conducted according to the tenets of the Declaration of Helsinki.

### 2.2. Statistical analyses

Statistical analysis was performed using Jamovi version 2.3.28. Continuous variables were expressed as mean (standard deviation (SD)) or median (Q1–Q3), depending on the data distribution. Categorical variables were presented as frequencies (n) and percentages (%). Categorical data were compared using Fisher’s exact test, and numerical data using the Student’s t-test or Mann–Whitney U test. A p-value of <0.05 was considered statistically significant.

## 3. Results

### 3.1. Characteristics of BD patients

Of the 65 patients in the study, 59 (90.8%) were male. The median (Q1–Q3) age at the time of diagnosis was 33.5 (26–40) years, while the median age at the time of diagnosis of arterial lesions was 38 (32–46) years. The median time between the BD diagnosis and the arterial event was 3 (1–10) years. The median follow-up duration at the participating centers (from first to last visit) was 4 (2–12.8) years, and disease duration from BD diagnosis was 142 (57–213) months. Of the total, 25 (39.7%) patients were smokers and four (6.3%) were former smokers. Venous thrombosis was present in 25 (43.1%) of the patients, mainly in the form of lower extremity deep vein thrombosis (DVT) (16 of 25; 64%). Budd–Chiari syndrome was present in three patients, while inferior vena cava thrombosis was detected in only one patient. Furthermore, 11 (17.2%) patients had ocular, eight (12.3%) had neurological, and five (7.8%) had gastrointestinal involvement. The clinical and demographic features of the patients are presented in Table 1.

### 3.2. Arterial features of BD

**Figure 1:**
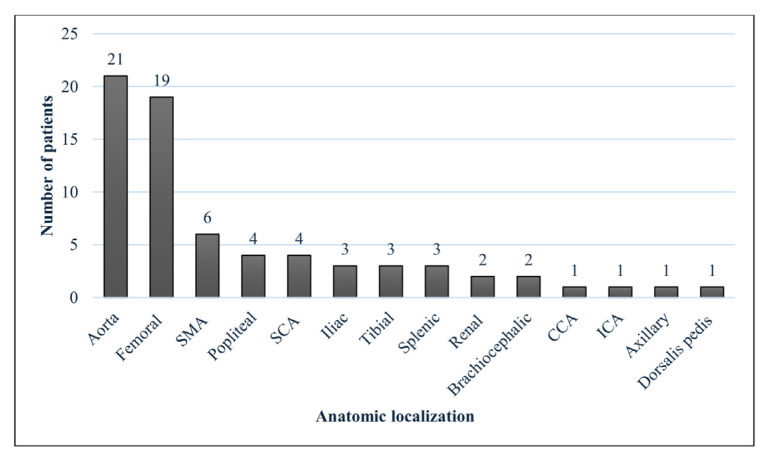
Anatomic localization of arterial lesions. Abbreviations: CCA, common carotid artery; ICA, internal carotid artery; SCA, subclavian artery; SMA, superior mesenteric artery.

**Figure 2:**
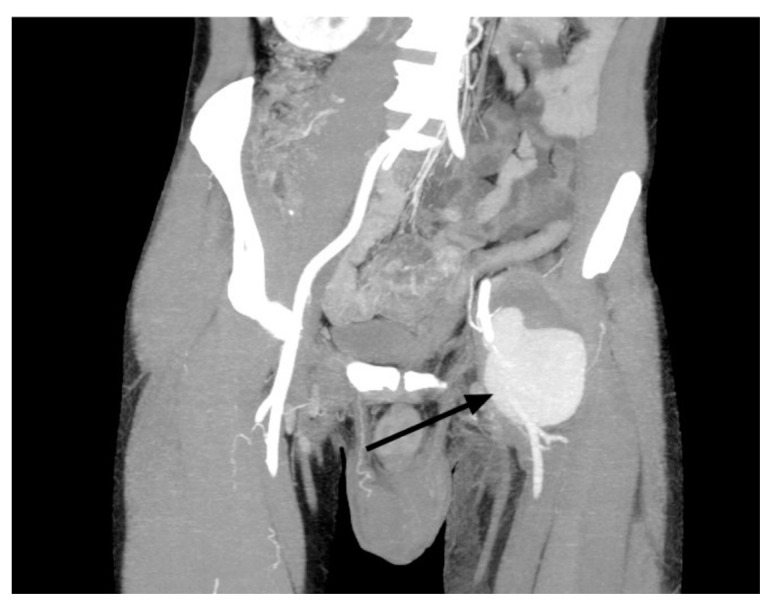
Femoral artery aneurysm in BD. CT angiography demonstrating a saccular aneurysm of the femoral artery (arrow).

**Figure 3:**
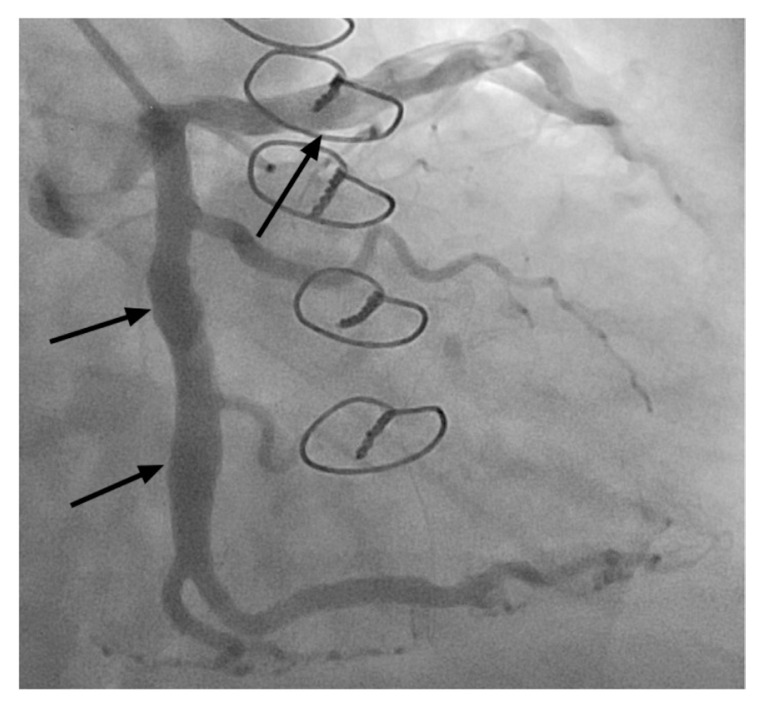
Coronary artery aneurysms in BD. Coronary angiography demonstrating multiple aneurysmal dilations of the coronary arteries (arrows). Sternal wires are visible, reflecting prior surgical intervention for Behçet’s disease–related aortic dissection.

The most commonly affected vessels were the aorta (n = 21, 32.3%) and the femoral artery (n = 19, 29.2%) (see Figure 1). Aneurysm (n= 50, 63.2%) and thrombosis (n= 18, 22.7%) were the predominant vascular lesion forms (percentages reflect lesion-based frequencies rather than mutually exclusive patient counts) (see Table 2a). A femoral artery aneurysm is shown in Figure 2. In addition to peripheral arterial involvement, pulmonary artery thrombosis was observed in eight patients, and a pulmonary artery aneurysm in a single patient. In addition, 11 (16.9%) patients had cardiac involvement (coronary artery aneurysms (n = 6); intracardiac thrombosis (n = 4); and aneurysm and thrombosis (n= 1). A representative coronary artery aneurysm is shown in Figure 3. Furthermore, 56 (86.2%) patients were symptomatic, with leg pain being the most common symptom, and seven of the nine asymptomatic patients had aortic involvement, detected incidentally (see Table 2b).

In univariate analyses, none of the evaluated clinical variables, including pathergy positivity (p = 1.000), venous involvement (p = 1.000), CRP (p = 0.762), ESR (p = 0.830), ocular involvement (p = 1.000), or neurological involvement (p = 0.421), showed a statistically significant association with aneurysmal arterial disease in patients with BD (see Supplementary Table).

### 3.3. Treatment

Pulse glucocorticoid (GC) therapy was initiated in 40 (61.5%) patients for 3 consecutive days and continued with a tapering GC regimen. Treatment was initiated with oral GC in 18 (27.6%) patients, with a median daily prednisone or equivalent dose of 40 (20–80) mg. The initial treatment data for seven patients were unavailable. The most commonly used IS therapies were cyclophosphamide (n = 43, 66.3%), azathioprine (n = 44, 67.7%), and tumor necrosis factor (TNF) inhibitors (adalimumab, n = 2; infliximab, n = 11; 20%). Fourteen (21.5%) patients experienced an arterial event while receiving IS therapy due to the involvement of another organ (azathioprine, n = 13; infliximab, n = 1).

Of the total, 39 (60%) patients underwent surgical and/or interventional procedures, and 32 (82%) of these procedures were successful. Two cases of pseudoaneurysm were documented in the available records; however, the true complication rate could not be reliably determined as postprocedural complications were not systematically recorded by all centers. Aside from two patients who underwent emergency surgery for aortic dissection, the remaining 30 patients with successful procedures had been receiving GC therapy prior to their interventions, while the two who underwent emergency surgery were started on GC and IS therapy postoperatively. Among the seven patients with unsuccessful procedures, six were recorded as receiving preprocedural GC and IS therapy, while the treatment data of the remaining patient were not available. The median interval between the arterial events and procedures was 15 (0–60) days. The most frequently performed procedures were graftings, followed by stent placements, and antiplatelet therapy was administered to all patients following these procedures. Twenty-four (36.9%) patients were placed on anticoagulant therapy following their arterial events (patients with aneurysm, n = 14; thrombosis, n = 7; aneurysm and thrombosis, n = 3). Among the patients who received anticoagulant therapy, 14 had concomitant venous and two had coronary artery involvement. No hemorrhagic complications were documented in the patients treated with anticoagulant therapy.

### 3.4. Follow-up

A second vascular event occurred in 10 (15.3%) patients during follow-up, four of which involved the same arterial region, while six occurred in different arterial regions. The median time from the first arterial event to relapse was 4.5 (2–6.75) years. Five patients had either discontinued IS therapy or used it only sporadically after the first arterial event. In the remaining five patients, the second arterial event occurred despite the continuing IS therapy (azathioprine, n = 3; infliximab, n = 2). At relapse, treatment was intensified in all patients, including high dose GC (n = 10), cyclophosphamide (n = 6), infliximab (n = 3), mycophenolate mofetil (n = 2), adalimumab (n = 1), and azathioprine (n = 1); while four patients underwent combination therapy. A third arterial event occurred in the same region in one patient receiving adalimumab, and in another in a different arterial territory under azathioprine therapy. Seven of the 39 patients (17.9%) who underwent surgical or interventional procedures and three out of the 26 patients (11.5%) who did not experienced at least one vascular event; but the difference was not statistically significant (p = 0.728). Similarly, seven of the 48 patients (14.6%) with arterial aneurysms and three of the 17 patients (17.6%) without aneurysms experienced at least one vascular event. Although the event rate was numerically higher in patients without aneurysms, the difference was neither statistically nor clinically significant and likely reflects random variation due to the limited sample size (p = 0.713).

At the last follow-up, 49 (75.3%) patients were receiving IS therapy, including azathioprine (n = 25), mycophenolate mofetil (n = 6), infliximab (n = 12), adalimumab (n = 3), and cyclophosphamide (n = 7); and nine were on combination therapy. Remission status data were available for 64 patients, indicating that 55 (85.9%) were in remission and nine (13.8%) still had active disease (evidenced by elevated acute phase reactants and vascular events detected through imaging). Mortality was observed in 6 (9.2%) patients (intracardiac thrombosis and pulmonary artery aneurysm: n = 1; acute coronary syndrome: n = 3 (all with coronary artery aneurysms); infection: n = 1; and alveolar hemorrhage, n = 1).

## 4. Discussion

This study has provided insights into the clinical spectrum and management of nonpulmonary arterial involvement in BD. The aorta and femoral arteries were the most commonly affected vessels, aneurysm was the predominant lesion type, and despite intensive treatment, relapse occurred in 15.3% of patients and mortality in 9.2%.

Arterial involvement in BD is rare, but is associated with high morbidity and mortality. As previously reported, major arterial involvement predominantly occurs in men and typically manifests late in the course of the disease, developing approximately 5 years after BD diagnosis [4]. In a cohort study of 882 patients with vascular BD, Tascilar et al. reported that patients with extrapulmonary arterial involvement were significantly older both at disease onset and at the time of vascular manifestation compared to those with other types of vascular involvement [10]. This is supported by the present study, in which the majority of patients were male and arterial involvement in vascular BD tended to occur at relatively older ages, despite the absence of a formal comparator group. Similarly, in a study by Saadoun et al. involving 101 BD patients with arterial involvement, the median time from diagnosis to the onset of arterial manifestations was 4 years, with most events occurring within 5 years of diagnosis [11]. In our cohort, this interval was comparable, with a median of 3 (1–10) years. Arterial lesions may be multiple and frequently coexist with venous thromboses [12]. Multiple and recurrent aneurysm formations have been reported in patients with BD. Previous studies estimate that approximately 30% of BD patients with arterial aneurysms develop multiple vascular lesions [13, 14]. In a study of 25 patients with nonpulmonary arterial involvement, Tüzün et al. reported venous thrombosis in 88% of the patients, with the majority of cases having DVT [15]. In the present study, 25 (43.1%) of the patients had venous thrombosis, primarily in the form of lower extremity DVT, and 13 (20%) exhibited multiple arterial aneurysms. This aligns with previous studies reporting that up to one-third of BD patients with arterial aneurysms develop multiple lesions. Our findings also revealed that peripheral arterial lesions frequently coexist with venous events, suggesting a broader overlap in vascular pathology. These data highlight the systemic and multifocal nature of vascular involvement in BD, and reinforce the need for comprehensive vascular imaging at diagnosis and during follow-up, particularly in patients presenting with signs of either arterial or venous disease [16].

Arterial involvement in BD has been reported to affect various vascular sites, including the aortic arch and the coronary, common carotid, subclavian, axillary, brachial, ulnar, renal, mesenteric, popliteal, tibial, cerebral, and pulmonary arteries [11,17–19]. Consistent with previous reports, aneurysm was the most frequent arterial lesion in our study population, predominantly affecting the aorta and femoral artery [12,15]. The initial pathological process in the affected arteries of BD is active arteritis, primarily involving the perivascular regions (vasa vasorum), and aneurysm formation is often painful due to its underlying inflammatory nature [20]. For this reason, peripheral aneurysms are more likely to be noticed by patients due to their superficial location, while aortic aneurysms may go unnoticed until complications occur. This was evident in our cohort, in which seven out of nine asymptomatic patients had aortic involvement. It has been previously reported that patients with arterial involvement may present with fever and laboratory evidence of systemic inflammation [11,21,22]. In light of this, arterial imaging should be strongly considered in BD patients who present with unexplained fever or unexplained elevations in acute-phase reactants in the absence of other clinical manifestations, as indicators of subclinical arterial involvement [16]. Imaging modalities such as CT angiography, MR angiography, Doppler ultrasonography, and 18-fluorodeoxyglucose-positron emission tomography are appropriate for evaluating arterial involvement. Given the risk of pathergy-related vascular complications, conventional angiography should be avoided unless absolutely necessary, and non-invasive imaging modalities should be preferred. Previous studies have reported the formation of aneurysms in BD following arterial puncture, surgery, or angiographic procedures [23,24].

In contrast to the findings of the present study, previous studies have reported a significant association between pathergy positivity and aneurysmal vascular involvement in patients with BD. Zhou et al. identified pathergy positivity as an independent risk factor for aneurysm development in a large cohort that included both pulmonary and systemic arterial aneurysms [23]. This discrepancy may be attributed to the limitations of our study, including its relatively small sample size, retrospective design, and the considerable proportion of missing data (pathergy status was unknown in 27% of patients), which may have reduced the ability of the study to detect a true association.

IS therapy is the cornerstone arterial disease management approach in BD [25], having been shown to reduce relapse rates and prolong survival in several retrospective studies [1,26]. The primary therapeutic approach for arterial involvement involves high-dose GCs and cyclophosphamide [27]. After remission is achieved, treatment typically continues with azathioprine, while a switch to TNF inhibitor therapy is recommended in refractory cases [1,28]. In the study conducted by Saadoun et al. involving 101 BD patients with arterial involvement, IS therapy was identified as the only factor significantly associated with remission. Patients treated with IS were four times more likely to achieve complete remission, and the annual incidence of arterial events significantly decreased following IS therapy [11]. In our cohort, high-dose GCs, often given in combination with cyclophosphamide, were the initial therapy in the majority of patients, in line with current treatment recommendations. At the time of the last follow-up, 75.3% of patients were under IS therapy, primarily azathioprine. Among those treated with IS, 81.6% were in remission.

The role of anticoagulation in BD remains controversial. In the study by Yıldızeli et al., pulmonary artery aneurysm was reported in only one of the nine BD patients who underwent pulmonary endarterectomy for chronic thromboembolic pulmonary hypertension, whereas thrombotic lesions predominated. In that series, all patients received anticoagulant therapy in the postoperative period in combination with IS treatment [29]. Saadoun et al. reported postoperative prosthetic thrombosis in 75% of patients without anticoagulation versus 40% with anticoagulation [11]. In a large French cohort of 807 patients, all cases with DVT (n = 296) received anticoagulation, including those with coexisting arterial aneurysms, and hemorrhagic complications were observed in only 2% [30]. In our cohort, 24 patients received anticoagulation, mostly due to concomitant venous (n =14) or coronary (n =2) involvement, while in eight cases the indication was undocumented; this should be regarded as a limitation inherent to the retrospective design of the present study. Although anticoagulation is generally discouraged in patients with arterial aneurysms due to rupture risk, our data suggest that clinicians often individualize treatment, particularly in the presence of concomitant venous thrombosis or cardiac involvement. In this respect, our study highlights the discrepancy between guideline-based recommendations and daily clinical practice.

Peripheral artery aneurysms that are large or symptomatic generally require urgent surgical intervention or stenting, while small, asymptomatic aneurysms may be managed with medical treatment. For both pulmonary and peripheral artery aneurysms, the choice of surgical intervention between graft insertion, ligation and bypass surgery can be made according to the size and location of the aneurysm and the surgeon’s experience [27]. Surgical or endovascular intervention alone is often insufficient for the management of arterial lesions in BD due to the underlying active vasculitis and the risk of postoperative complications such as graft occlusion or anastomotic pseudoaneurysm. Several studies have underlined the essential role of IS therapy both before and after vascular procedures in controlling disease activity and reducing the risk of recurrence. In their series on aortic pseudoaneurysm repairs, Balcıoğlu et al. identified favorable long-term outcomes with endovascular stent grafting, but only when combined with both preoperative and postoperative IS therapy, and reported no aneurysm recurrence during a mean follow-up of 40 months [31]. Similarly, Koo et al. reported recurrence and stent thrombosis exclusively in patients who had discontinued IS therapy after endovascular repair [32]. Kalko et al. reported that BD patients who achieved remission prior to surgery had a lower incidence of postoperative complications [33]. In our cohort, 60% of patients underwent surgical or interventional procedures, primarily grafting, followed by stent placement. While 82% of these procedures were successful, the treatment failed in seven patients. Notably, six of the seven patients with unsuccessful procedures had received preprocedural GC and IS agents, suggesting that although medical therapy is essential, it may not always ensure procedural success, particularly in cases with active inflammation or technical challenges. This suggests that more aggressive treatments such as TNF inhibitors may help procedural success. In contrast, two patients who underwent emergency surgery for aortic dissection did not receive IS therapy prior to intervention, but were started on GC and IS therapy postoperatively, and their procedures were successful. These cases emphasize the potential benefits of prompt surgical intervention followed by the timely initiation of IS therapy.

The relapse rate in our cohort was 15.3%, consistent with previous reports of up to 20% [1]. Relapses occurred at both previously involved and unaffected sites, and half were linked to irregular or discontinued IS therapy, while the other half occurred despite ongoing treatment. In Türkiye, relapse rates of venous thrombosis have been reported as 45% in a prospective cohort and 32.9% in a retrospective cohort [26,34]. Our relapse rate (15.3%) was numerically lower; however, direct comparisons with venous cohorts should be interpreted with caution due to methodological differences. While arterial involvement in BD is generally associated with more severe outcomes, the risk of relapse may differ across vascular phenotypes.

Previous studies have reported relatively low mortality rates in BD patients with nonpulmonary aneurysms, although the recurrence rates remain high [23]. A significant decrease in mortality rate has been reported in such patients, from 17% to 5%, in time [15,20]. The mortality rate in our cohort was 9.2%, predominantly related to cardiac involvement.

There are several limitations to our study. Its retrospective design may have introduced selection and information bias, as data were collected from existing medical records. Furthermore, although this was a multicenter study, the relatively small sample size limits the generalizability of the findings. In addition, treatment decisions were not standardized across centers, being left to the discretion of individual physicians, further limiting comparisons of outcomes.

Arterial involvement in young patients without significant atherosclerotic risk factors should raise suspicion for BD. Nonpulmonary arterial lesions may represent the initial manifestation and are frequently misattributed to other vascular conditions, leading to delays in diagnosis and appropriate treatment. Suspicions should be raised when multiple arterial territories are involved, when arterial and venous lesions coexist, or when the clinical course is atypical, such as rapid progression or recurrence after vascular intervention. The presence of concomitant systemic features, including recurrent oral or genital ulcers, uveitis, or unexplained venous thrombosis, further supports diagnosis. Early recognition is essential, as optimal management requires the prompt initiation of IS therapy in addition to vascular intervention to reduce complications, to prevent disease progression and to reduce the risk of relapse.

In conclusion, arterial involvement in BD mostly presents with aneurysm formation, especially in male patients. Around 50% of patients require surgical interventions in addition to IS therapy. Some 15.3% of patients relapsed during follow-up, indicating that recurrent arterial events are not uncommon, underlining the need for close long-term follow-up and sustained IS treatment. Although lower than the rates reported in venous disease, direct comparisons should be made with caution. The 9.2% of patients who succumbed to the disease tended to have both arterial and cardiac involvement.

## Supplementary material

**Table:** Univariate comparison of patients with and without aneurysmal arterial disease

Characteristic	Aneurysm (+) (n= 48 *)	Aneurysm (−) (n= 17)	p-value
Pathergy positivity **, **n (%)**	20 (60.6%)	9 (64.3%)	1.000
Venous involvement **, **n (%)**	19 (43.2%)	6 (42.9%)	1.000
**Ocular involvement, n (%)**	8 (16.7%)	3 (17.6%)	1.000
**Neurological involvement, n (%)**	5 (10.4%)	3 (17.6%)	0.421
**C-reactive protein (CRP), mg/L, median (Q1–Q3)**	24 (10–88)	56.5 (12.8–92)	0.762
**Erythrocyte sedimentation rate (ESR), mm/hr, median (Q1–Q3)**	39.5 (21–67)	43.5 (31–54.3)	0.830

* Aneurysm status was analyzed on a patient basis.

** Missing data were excluded from the analyses.

## Figures and Tables

**Table 1:** Baseline clinical characteristics of patients.

Patient characteristics	(N = 65)

**Sex, n (%)**	
** ** **Male**	59 (90.8%)
** ** **Female**	6 (9.2%)

**Age onset, years (median)**	33.5 (26–40)

**Age at first arterial event, years (median)**	38 (32–46)

**Follow-up time, years (median)**	4 (2–12.8)

**Smoking, n (%)**	25 (39.7%)

**Oral ulcer, n (%)**	62 (95.4%)

**Genital ulcer, n (%)**	41 (63.1%)

**Erythema nodosum, n (%)**	22 (33.8%)

**Papulopustular lesions, n (%)**	27 (41.5%)

**Pathergy positivity, n (%)**	29 (44.6%)

**Arthritis, n (%)**	10 (15.4%)

**Venous involvement, n (%)**	25 (43.1%)

**Ocular involvement, n (%)**	11 (17.2%)

**Neurological involvement, n (%)**	8 (12.3%)

**Gastrointestinal involvement, n (%)**	5 (7.8%)

**Table 2a:** Arterial lesion types and sites in 65 patients with BD.

Parameter	N (%)

**Type of arterial lesion (n = 79)**	
Aneurysm	50 (63.2%)
Thrombosis	18 (22.7%)
Stenosis	7 (8.8%)
Aortitis	2 (2.5%)
Dissection	2 (2.5%)

**Arterial site**	
Isolated	52 (80%)
Multiple	13 (20%)

**Table 2b:** Initial clinical presentation and inflammatory markers in patients with nonpulmonary arterial involvement of BD.

Parameter	N (%)

**Signs and symptoms at presentation**	
** ** **Leg pain**	23 (35.3%)
** ** **Abdominal pain**	15 (23.1%)
** ** **Chest/back pain**	10 (15.3%)
** ** **Dyspnea**	5 (7.6%)
** ** **Fever**	3 (4.6%)
** ** **Mass in the neck**	1 (1.5%)
** ** **Syncope**	1 (1.5%)
** ** **Asymptomatic**	9 (13.8%)

**C-Reactive protein level (mg/L), median**	25 (10–88)

**Erythrocyte sedimentation rate (mm/h), median**	41 (21–64)

## Data Availability

Data supporting the findings of this study can be obtained from the corresponding author [Özge Karakök] upon reasonable request.
